# Comparison of indoor contact time data in Zambia and Western Cape, South Africa suggests targeting of interventions to reduce *Mycobacterium tuberculosis* transmission should be informed by local data

**DOI:** 10.1186/s12879-016-1406-5

**Published:** 2016-02-09

**Authors:** Nicky McCreesh, Clare Looker, Peter J. Dodd, Ian D. Plumb, Kwame Shanaube, Monde Muyoyeta, Peter Godfrey-Faussett, Elizabeth L. Corbett, Helen Ayles, Richard G. White

**Affiliations:** 1 TB Modelling Group, Department of Infectious Disease Epidemiology, London School of Hygiene and Tropical Medicine, London, UK; 2 Health Economics and Decision Science, School of Health and Related Research, University of Sheffield, Sheffield, UK; 3 ZAMBART Project, School of Medicine, University of Zambia, Lusaka, Zambia; 4 TB Department, Centre for Infectious Disease Research in Zambia, Lusaka, Zambia; 5 Faculty of Infectious and Tropical Diseases, London School of Hygiene and Tropical Medicine, London, UK; 6 HIV and TB Theme, Malawi Liverpool Wellcome Trust Clinical Research Programme, Blantyre, Malawi

**Keywords:** Tuberculosis, South Africa, Zambia, Contact data, Transmission, *Mycobacterium tuberculosis*

## Abstract

**Background:**

In high incidence settings, the majority of *Mycobacterium tuberculosis* (*M.tb*) transmission occurs outside the household. Little is known about where people’s indoor contacts occur outside the household, and how this differs between different settings. We estimate the number of contact hours that occur between adults and adult/youths and children in different building types in urban areas in Western Cape, South Africa, and Zambia.

**Methods:**

Data were collected from 3206 adults using a cross-sectional survey, on buildings visited in a 24-h period, including building function, visit duration, and number of adults/youths and children (5–12 years) present. The mean numbers of contact hours per day by building function were calculated.

**Results:**

Adults in Western Cape were more likely to visit workplaces, and less likely to visit shops and churches than adults in Zambia. Adults in Western Cape spent longer per visit in other homes and workplaces than adults in Zambia. More adults/youths were present at visits to shops and churches in Western Cape than in Zambia, and fewer at homes and hairdressers. More children were present at visits to shops in Western Cape than in Zambia, and fewer at schools and hairdressers. Overall numbers of adult/youth indoor contact hours were the same at both sites (35.4 and 37.6 h in Western Cape and Zambia respectively, *p* = 0.4). Child contact hours were higher in Zambia (16.0 vs 13.7 h, *p* = 0.03). Adult/youth and child contact hours were highest in workplaces in Western Cape and churches in Zambia. Compared to Zambia, adult contact hours in Western Cape were higher in workplaces (15.2 vs 8.0 h, *p* = 0.004), and lower in churches (3.7 vs 8.6 h, *p* = 0.002). Child contact hours were higher in other peoples’ homes (2.8 vs 1.6 h, *p* = 0.03) and workplaces (4.9 vs 2.1 h, *p* = 0.003), and lower in churches (2.5 vs 6.2, *p* = 0.004) and schools (0.4 vs 1.5, *p* = 0.01).

**Conclusions:**

Patterns of indoor contact between adults and adults/youths and children differ between different sites in high *M.tb* incidence areas. Targeting public buildings with interventions to reduce *M.tb* transmission (e.g. increasing ventilation or UV irradiation) should be informed by local data.

**Electronic supplementary material:**

The online version of this article (doi:10.1186/s12879-016-1406-5) contains supplementary material, which is available to authorized users.

## Background

Globally, tuberculosis (TB) incidence is falling by only 1–2 % per year [1]. With current control efforts, targets for TB control will not be met [2]. Supplementing ‘case finding’ based control measures with ‘place finding’ based methods is a promising approach for reducing incidence [3], but is currently hampered by a poor understanding of where *Mycobacterium tuberculosis* (*M.tb*) transmission occurs in high incidence settings.

*M.tb* is a droplet nuclei infection, with probability of infection related to duration of exposure to infected air [4]. Both ventilation and ultraviolet light greatly reduce concentrations of viable droplet nuclei [5], limiting transmission outdoors. Studies in sub-Saharan Africa suggest that only a small proportion of transmission occurs between household members [6, 7]. It is therefore highly probable that the majority of infection occurs indoors, in buildings other than individual’s own homes. Individual outbreaks of *M.tb* infection have been linked to a wide range of settings, including public transport [8, 9], schools [10], churches [11, 12] and healthcare facilities [13, 14]. These kinds of study provide some information on locations where transmission *can* occur, but as outbreak investigations are typically only undertaken when unusual patterns of disease are noticed, they give little insight into the types of location where transmission is most common.

One way of improving our understanding of potential transmission locations is to collect and analyse comprehensive data on people’s movements, activities, and contacts, over the course of a defined period of time. One study in South Africa, which collected activity data using a 24-h diary, found that crèche/school, workplaces, and other people’s houses accounted for 74 % of indoor contact hours that occurred outside respondents’ own houses [15, 16]. Patterns of movement are likely to be different in different settings however, having an impact both on where *M.tb* transmission occurs, and on the most effective places to target interventions to reduce transmission. In this study, we compare for the first time patterns of indoor contact in different settings. We do this using data from two urban and peri-urban, high incidence sub-Saharan African settings: communities in the Western Cape province of South Africa, and communities in Zambia. We focus on contacts involving adults, as children as generally less infectious than adults, and therefore contact with adults with TB is responsible for the majority of *M.tb.* infection in both adults and children [17].

## Methods

### Survey methods

The sampling frame for this study was adults (≥18 years) enrolled in the ZAMSTAR [18] final TB prevalence survey conducted in 2010 in 16 communities in Zambia and 8 communities in the Western Cape, South Africa. The 2010 TB prevalence survey recruited between 4000 and 5000 individuals per community by visiting all households in randomly selected standard enumeration areas (SEAs). This study consisted of a subsequent cross-sectional face-to-face interview survey of TB prevalence survey enrolees that took place in February and March 2011 in Zambia, and in May and July 2011 in Western Cape. Four SEAs from each ZAMSTAR community were randomly selected proportional to size, and within each SEA ten individuals were randomly selected from four age and gender strata: men aged 18–29 years, men aged ≥30 years, women aged 18–29 years, and women aged ≥30 years (160 per community). Individuals were not eligible if they had not spent the previous night in the SEA or did not provide informed consent. If an individual was ineligible or was not found after two visits, another individual was randomly selected from the same stratum in that SEA. Recruitment was planned to continue until 10 individuals per SEA were selected within each stratum.

Interviews were carried out in participants’ homes by trained field staff using a standardized questionnaire that was piloted in Zambia, following a qualitative survey in Zambia that rapidly gathered data on places of significance to TB transmission, children’s space and popular knowledge of TB transmission [19]. Interviewees were asked to list buildings (other than their own home) that they had entered the day before the interview (from midnight to midnight). Buildings were considered to be enclosed areas with walls and a roof, excluding transport. For each building they listed, they were asked:

- What type of building did you enter? (other home, shop, church, bar/disco/shebeen, school, clinic/hospital, hairdresser/barber, own work building, other).
- How much time did you spend in total inside this building? (less than 5 min, 5–10 min, 11–59 min, 1–4 h, 5–8 h, 9–13 h, more than 14 h).
- How many adults and youths (those older than 12) were present? (fewer than 5, 5–9, 10–20, more than 20).
- How many children (5–12) were present? (fewer than 5, 5–9, 10–20, more than 20).

Further details of the sampling and interview methods, and an English version of the study questionnaire are available in Dodd et al 2015 [20].

### Data analysis

Data were double-entered into an SQL Server database and analysed using Stata [21]. Data from two rural communities in Zambia were excluded (leaving data from 14 communities in Zambia), as only urban communities were surveyed in Western Cape. All results were weighted for community population size and the age and gender proportions in the SEA. This approach allowed for the two-stage, stratified sample design and corrected for differences between sample and community demography, yielding estimates that apply to randomly chosen individuals from survey communities. Within each site (Zambia and Western Cape), estimates were also weighted by the day of the week that participants were asked about (weekend or weekday), as interview numbers varied by day of week (Additional file 1: Table S1), and activity patterns are likely to vary by day of week. For visits to churches only, a sensitivity analysis was conducted where estimates were weighted according to whether the participants had been asked about their building visits on Sunday or on any other day of the week.

Mean numbers of adult and child contact hours were calculated for each person and type of building by multiplying the time spent in the building by the number of adults and children present. Where an individual reported visiting two or more buildings of the same type, the number of contact hours were summed over all visits. Individuals who did not report visiting a particular type of building were assigned zero adult and child contact hours for buildings of that type. As durations of building visits and numbers of adults/youths and children present were recorded categorically, category mid-points were used in calculations (e.g. a duration of 7.5 min was used for reported visits of 5–10 min). A duration of 14 h was used for visits that were reported to have lasted more than 14 h. Visits where more than 20 adults/youths or children were reported to have been present were assigned a value of 30. As the lowest category for number of adults/youths or children present contained zero, and as the highest, open-ended category was frequently reported (Additional file 1: Figures S1 and S2), a sensitivity analysis was conducted to explore the effect of the choice of values assigned to categories. Two additional estimates of numbers of adults/youths and children present were calculated: ‘Low’, where the lower bounds of categories were used (0 for ‘fewer than 5’, 5 for ‘5–9’, 10 for ‘10–20’, and 21 for ‘more than 20’), and ‘High’, where upper bounds were used (4 for ‘fewer than 5’, 9 for ‘5–9’, 20 for ‘10–20’, and 50 for ‘more than 20’).

A completed Strobe checklist for this paper is available in the additional files (Additional file 2).

### Ethics

Ethics approval was obtained from University of Stellenbosch Health Research Ethics Committee (N04/10/173), University of Zambia Biomedical Research Ethics Committee (007-10-04), and London School of Hygiene and Tropical Medicine Ethics Committee (A211 3008).

## Consent

Research assistants read through an interview sheet and consent form with the participants, and participants were given the opportunity to ask questions. Participants then gave written consent. Participants were given a copy of the documents to keep. The wording of the interview sheet and consent form was approved by the three ethics committees that reviewed the study.

## Results

A total of 3227 adults living in urban areas were interviewed. 21 did not provide information on buildings visited, and were excluded from the analysis. Results are therefore presented from 3206 adults: 1270 (40 %) from Western Cape, South Africa, and 1941 (60 %) from Zambia (Table 1). 1661 (52 %) respondents were female, and 1550 (48 %) were male. Further details of the study participants are given in Dodd et al*.* [20]. Data were available on 2766 building visits. Of these, 29 were missing data on building type, 38 on visit duration, 73 on the number of adults/youths present, and 217 on the number of children present. 289 building visits were missing data on any of the four variables. Building visits were excluded from analyses when relevant data were missing. All subsequent results are weighted using the method described above.

**Table 1:** Summary of reported building visits, by building type and country

		Western Cape	Zambia	*p*-value
Proportion of respondents who left their home		56 % (51 % - 61 %)	59 % (53 %–64 %)	0.5
Number of buildings visited yesterday (if left home**)**	0	0.90 % (0.23 %–3.5 %)	3.6 % (1.9 %–6.7 %)	**<0.0001**
	1	82 % (77 %–86 %)	58 % (51 %–65 %)	
	2	14 % (10 %–18 %)	26 % (22 %–31 %)	
	3+	3.8 % (2.3 %–6.0 %)	12 % (9.5 %–16 %)	
Proportion of respondents who visited:	Other homes	23 % (20 %–26 %)	19 % (16 %–23 %)	0.2
	Shops	8.2 % (5.8 %–12 %)	19 % (16 %–22 %)	**<0.0001**
	Churches	5.2 % (3.3 %–8.1 %)	12 % (9.3 %–15 %)	**0.001**
	Bars	*2.3 % (1.4 %–3.8 %)*	7.3 % (5.6 %–9.5 %)	*
	Schools	3.7 % (2.7 %–5.1 %)	4.1 % (3.2 %–5.2 %)	0.7
	Clinics	1.6 % (1.0 %–2.5 %)	2.8 % (1.9 %–3.9 %)	0.07
	Hairdressers	0.70 % (0.34 %–1.5 %)	1.5 % (0.92 %–2.3 %)	0.08
	Work	16 % (13 %–19 %)	6.3 % (5.1 %–7.8 %)	**<0.0001**
	Other	3.7 % (2.5 %–5.3 %)	8.4 % (6.4 %–11 %)	**0.001**
Mean visit duration (hours) to:	Other homes	3.5 (2.9–4.2)	2.4 (1.8–2.9)	**0.009**
	Shops	1.2 (0.84–1.5)	0.86 (0.61–1.1)	0.1
	Churches	2.7 (2.3–3.0)	2.8 (2.5–3.0)	0.8
	Bars	*3.6 (2.5–4.6)*	3.0 (2.6–3.4)	
	Schools	4.5 (3.6–5.4)	4.6 (3.8–5.5)	0.8
	Clinics	3.5 (2.3–4.7)	2.4 (1.9–2.8)	0.09
	Hairdressers	3.7 (1.7–5.8)	2.5 (1.4–3.6)	0.3
	Work	7.1 (6.7–7.5)	7.0 (6.0–8.0)	0.9
	Other	4.0 (2.6–5.3)	2.3 (1.7–2.9)	**0.03**
Mean number of adults/youths present per visit to:	Other homes	4.0 (3.3–4.8)	5.5 (4.6–6.4)	**0.01**
	Shops	16 (12–19)	8.4 (6.8–10)	**0.0002**
	Churches	27 (24–29)	24 (23–25)	**0.05**
	Bars	*20 (16–23)*	22 (20–24)	*
	Schools	24 (21–28)	25 (22–28)	0.8
	Clinics	26 (23–30)	23 (20–25)	0.1
	Hairdressers	3.9 (1.8–6.1)	8.1 (4.9–11)	**0.04**
	Work	14 (12–16)	15 (12–18)	0.5
	Other	17 (13–22)	13 (11–15)	0.1
Mean number of children present per visit to:	Other homes	2.9 (2.4–3.4)	3.2 (2.7–3.8)	0.4
	Shops	8.6 (5.8–11)	4.8 (3.7–5.9)	**0.01**
	Churches	18 (12–24)	18 (15–20)	0.8
	Bars	*3.2 (1.6–4.7)*	3.6 (2.9–4.3)	*
	Schools	3.9 (2.2–5.7)	8.4 (5.3–11)	**0.01**
	Clinics	19 (13–24)	16 (12–19)	0.4
	Hairdressers	2.0**	3.7 (2.1–5.2)	**0.04**
	Work	4.4 (3.3–5.5)	5.1 (3.4–6.6)	0.5
	Other	4.8 (2.2–7.5)	7.4 (5.2–9.6)	0.1

*p*-values in bold indicate significance at the 95 % level. All values are weighted, as described in the methods. *Estimates for bars in Western Cape are considered to be unreliable (see discussion), and therefore *p*-values are not shown. ** Confidence intervals could not be calculated due to low numbers

There was no difference between countries in the proportion of adults who reported leaving their house the day before the interview (Table 1. Zambia: 56 % (95 % CI 51–61 %), Western Cape: 59 % (95 % CI 51–66 %), *p* = 0.6). Amongst adults who left their house, adults living in Zambia were more likely to visit a higher number of other buildings than adults living in Western Cape (*p* < 0.0001). 26 % (95 % CI 22–31 %) and 12 % (95 % CI 10–16 %) of respondents from Zambia reported visiting two or more than two buildings respectively, compared to only 14 % (95 % CI 10–18 %) and 4 % (95 % CI 2–6 %) in Western Cape.

The most commonly visited buildings by adults in Western Cape were other people’s houses (23 %, 95 % CI 20–26 %) and workplaces (16 %, 95 % CI 13–19 %) (Table 1). In Zambia, the most commonly visited buildings were other people’s homes (19 %, 95 % CI 16–23 %) and shops (19 %, 95 % CI 16–22 %). Compared to respondents from Zambia, respondents from Western Cape were more likely to have visited workplaces (16 % vs 6 %, *p* < 0.0001), and less likely to have visited shops (8 % vs 19 %, *p* < 0.0001), churches (5 % vs 12 %, *p* = 0.001), and buildings classed as ‘other’ (4 % vs 8 %, *p* = 0.001).

In both settings, mean visit durations were highest for workplaces (7.0 h in both settings) and schools (Western Cape: 4.5 h, Zambia: 4.6 h) (Table 1). Compared to respondents from Zambia, respondents from Western Cape spent longer per visit on average in other people’s homes (3.5 h vs 2.4 h, *p* = 0.009) and buildings classed as ‘other’ (4.0 h vs 2.3 h, *p* = 0.03). The distribution of reported time by building type and site is shown in Additional file 1: Figure S3.

In both settings, the mean number of adults/youths reported per visit was highest in churches (Western Cape: 27, Zambia: 24), schools (Western Cape: 24, Zambia: 25), and clinics (Western Cape: 26, Zambia: 23) (Table 1). The mean number of children reported per visit was highest in churches (Western Cape: 18 Zambia: 18), and clinics (Western Cape: 19, Zambia: 16). While the sensitivity analysis altered estimates of the absolute number of adults/youths and children present per building type and site, it had little effect on the relative differences between different building types (Additional file 1: Table S2). Compared to respondents from Zambia, respondents from Western Cape reported more adults/youths present per visit on average for visits to shops (16 vs 8.4, *p* = 0.0002) and churches (27 vs 24, *p* = 0.05), and fewer present for visits to other homes (4.0 vs 5.5, *p* = 0.01) and hairdressers (3.9 vs 8.1, *p* = 0.04). They reported more children present per visit for visits to shops (8.6 vs 4.8, *p* = 0.01), and fewer present per visit for visits to schools (3.9 vs 8.4, *p* = 0.01) and hairdressers (2.0 vs 3.6, *p* = 0.04). The sensitivity analysis showed that assumptions made about number of adults/youths or children present had some effect on the results, with some differences considered significant at the 95 % level in the main analysis but not the sensitivity analysis. The choice of threshold for significance was arbitrary however, and for all building types, where substantially more adults/youth or children were estimated to be present per visit in Western Cape compared to Zambia in the main analysis, more were also estimated to be present in the sensitivity analysis (and *vice versa*). Similarly, where there was little difference between countries in the estimated mean number of adults/youths or children present in the main analyses, there was little difference in the sensitivity analysis.

Overall, there was no difference between Western Cape and Zambia in the total mean number of adult/youth contact hours per day (Table 2. Western Cape: 35 h, Zambia: 38 h, *p* = 0.4). There was a small difference in the reported mean number of child contact hours per day, with adults in Western Cape reporting a mean of 14 contact hours compared to 16 in Zambia (*p* = 0.03). Compared to respondents in Zambia, respondents in Western Cape reported more adult/youth contact hours on average in workplaces (15 vs 8.0, *p* = 0.004), and fewer in churches (3.7 vs 8.5, *p* = 0.002). They also reported more child contact hours on average in other homes (2.8 vs 1.6, *p* = 0.03) and workplaces (4.9 vs 2.1, *p* = 0.003), and fewer in churches (2.5 vs 6.2, *p* = 0.004) and schools (0.39 vs 1.5, *p* = 0.01).

**Table 2:** Adult/youth and child contact hours per adult per day, by building type and country

		Western Cape	Zambia	*p*-value
Mean number of adult/youth contact hours per adult per day in:	Other homes	3.6 (2.3–4.9)	3.2 (1.8–4.5)	0.6
	Shops	2.6 (1.0–4.1)	2.5 (1.4–3.7)	1.0
	Churches	3.7 (1.9–5.4)	8.6 (6.1–11)	**0.002**
	Bars	*2.0 (0.7–3.2)*	5.3 (3.8–6.9)	*
	Schools	4.7 (3.1–6.4)	5.1 (3.5–6.7)	0.7
	Clinics	1.5 (0.72–2.3)	1.7 (0.93–2.4)	0.8
	Hairdressers	0.14 (0.00–0.32)	0.43 (0.040–0.81)	0.2
	Work	15 (11–19)	8.0 (5.3–11)	**0.004**
	Other	2.1 (0.62–3.6)	2.9 (1.6–4.2)	0.4
	Total	35 (29–41)	38 (32–43)	0.4
Mean number of child contact hours per adult per day in:	Other homes	2.7 (1.8–3.7)	1.6 (0.99–2.1)	**0.03**
	Shops	1.4 (0.51–2.2)	1.2 (0.67–1.7)	0.7
	Churches	2.5 (1.0–4.0)	6.2 (4.2–8.1)	**0.004**
	Bars	*0.22 (0.093–0.35)*	0.67 (0.44–0.90)	*
	Schools	0.39 (0.26–0.52)	1.5 (0.63–2.4)	**0.01**
	Clinics	1.0 (0.37–1.7)	1.1 (0.51–1.7)	0.8
	Hairdressers	0.053 (0.00093–0.10)	0.23 (0.00–0.47)	0.2
	Work	4.9 (3.3–6.4)	2.1 (1.2–3.1)	**0.003**
	Other	0.52 (0.24–0.80)	1.6 (0.56–2.5)	0.1
	Total	14 (11–16)	16 (13–19)	**0.03**

*p*-values in bold indicate significance at the 95% level. All values are weighted, as described in the methods. * Estimates for contact hours in bars in Western Cape are considered to be unreliable (see discussion), and therefore *p*-values are not shown

The sensitivity analysis showed that results for contact hours with adults/youths were robust to assumptions made in converting ‘number of adults present’ from a categorical to a continuous variable (Additional file 1: Table S3). A small number of the findings for children were less robust. In particular, there was no evidence for any difference between the sites in the mean total number of child contact hours in the ‘High’ estimates, although a lower number of child contact hours were still estimated for Western Cape (24 vs 26, *p* = 0.4). In addition, in the ‘Low’ estimates, there was a significant difference between countries in the number of contact hours in buildings classed as ‘other’, and no significant difference in the number of contact hours in other homes and at workplaces.

**Fig. 1:**
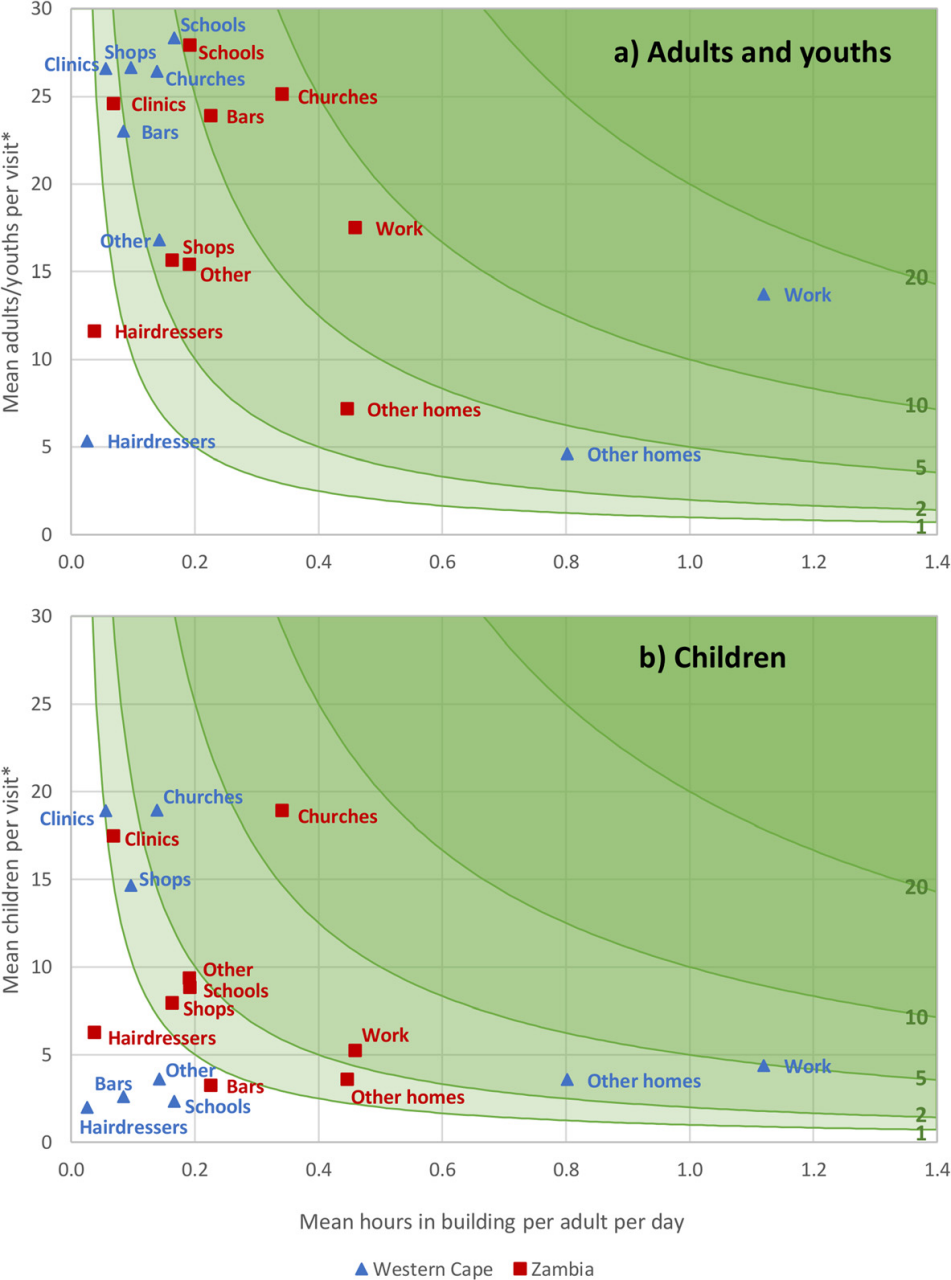
Reported contact hours per adult per day, by building type and site. Mean number of total contact hours with **a** adults and youths and **b** children, in each building type per adult per day, by site. This is product of (x axis) the mean number of hours in the building type per adult per day and (y axis) mean number of adults/youths or children present per visit. Green lines indicate contours of 1, 2, 5, 10, and 20 mean total contact hours with adults/youths or children, per adult per day. *Mean number of adults/youths and children per visit is weighted according to visit duration. Values therefore differ slightly from those presented in Table 1

In the majority of cases, where there was a difference between sites in the mean number of reported adult/youth or child contact hours, the difference was largely due to a difference in the mean amount of time spent in the building per day (Fig. 1). The exception to this was child contact time in schools, where the number of contact hours estimated for Zambia was higher as a result of more children being present per visit on average. For some other building types, differences in the number of hours spent in the building per day and the mean number of adults/youths or children present largely cancelled each other out, resulting in there being little difference between sites in overall contact hours. For instance, the reported mean visit duration to other homes was 3.5 h in Western Cape and 2.4 h in Zambia (*p* = 0.009), and the mean number of adults/youths present per visit was 4.0 in Western Cape and 5.5 in Zambia (*p* = 0.01). When combined, these figures resulted in there being little difference between sites in the mean daily number of contact hours in other homes (Western Cape: 3.6 contact hours, Zambia: 3.2 contact hours, *p* = 0.6). This ‘cancelling out’ also occurs to some extent for both adult/youth and child contacts in shops.

Weighting by Sunday or any other day (as opposed to weekday or weekend) slightly reduced estimates of both adult/youth and child contact hours in churches for both sites, but had little effect on the differences between the two countries (Additional file 1: Table S4).

## Discussion

In this study, we compare for the first time the contribution of different building types to indoor contact time between different urban, high TB incidence settings. We show that indoor contact hours between adults and both adults/youths and children (aged 5–12 years) were highest in workplaces in Western Cape, South Africa, and in churches in Zambia. In both workplaces and churches, differences between the two sites were largely due to differences in the mean amount of time spent in the buildings per day, rather than differences in the number of people present in the buildings.

In Western Cape, 43 % of all reported indoor adult/youth contact hours (outside people’s own homes) occurred in workplaces. Targeting these buildings with interventions to reduce transmission, such as improving ventilation, adding UV irradiation lighting, or workplace contact tracing, may therefore be a very effective way to reduce *M.tb.* incidence in adults in Western Cape. More surprisingly, 36 % of all reported contact hours between adults and children also occurred in workplaces, implying that any intervention in workplaces could also substantially reduce infection incidence in children. In Zambia, workplaces accounted for 21 % and 13 % of all reported indoor contact hours with adults/youths and children respectively. Interventions in workplaces may therefore have less effect on infection incidence in Zambia than in Western Cape. In contrast, interventions targeting churches may be more effective in reducing incidence in Zambia, particularly in children, as 23 % and 38 % of reported contact hours with adults/youths and children respectively occurred in churches.

Our results demonstrate the importance of considering local context when attempting to identify sites where *M.tb* transmission may occur, and when designing interventions to reduce incidence. Patterns of contact may vary greatly between different countries, and are also likely to vary within countries (e.g. between urban and rural locations). Spending times in bars [22] and health care facilities [14] are often considered to be risk factors for TB disease. Spending time in churches and having an indoor workplace may also be important risk factors in some settings.

Future contact data collection should also distinguish between numbers of new and repeat contacts by location. All else being equal, transmission probability will increase with increasing contact time. There is considerable variation between people in susceptibility to *M.tb.* infection and TB disease however, and in infectiousness given active disease. Chance contacts between highly susceptible and infectious people may therefore be important to overall transmission, and infection risk may be higher than we have estimated in locations where cumulative numbers of new contacts are high. This may also help to explain why only a small proportion of people are infected by people living in their own households, despite the large amount of time people spend in their own houses.

There are four main limitations to our study. The first is that no data were collected on building ventilation and crowding. Contacts that occur in close proximity in badly ventilated buildings are likely to be associated with a higher risk of *M.tb* transmission than contacts in less crowded, better ventilated buildings. The suitability of buildings for transmission may also vary between different settings, and therefore differences in transmission risk in, say, churches between Zambia and South Africa may not be the same as differences in contact time. Bars and churches have previously been identified as locations that can be very favourable for *M.tb* transmission in South Africa, due to high densities of people and poor ventilation [23]. A better understanding of the nature of indoor workplaces in high TB, sub-Saharan African settings would improve estimates of their likely contribution to *M.tb* transmission.

Secondly, no data were collected on the prevalence of people with pulmonary and smear positive TB in different building types. While our findings show that few contact hours occur in clinics in either location, this is likely to underestimate the importance of these locations for transmission, as it is very probable that a far higher proportion of contacts in clinics will have TB disease than contacts in other settings. This means, for example, that both adults/youths and children may be exposed to more potentially infective person contact hours on average in clinics than in churches, if prevalences of pulmonary TB in people present at clinics in Zambia are more than five times higher than prevalences in people present in churches. In addition, people attending clinics may be more susceptible to infection and/or progression to disease. The social mixing patterns of people with symptomatic TB may also be different in other ways, for instance people who are very ill may spend less time in bars. This would reduce the contribution of bars to overall transmission.

Thirdly, very few data were collected in Western Cape on building visits that occurred on Fridays and Saturdays. To correct for this bias, the data were weighted by day of week (weekday or weekend). This approach is valid, provided that building visits and contacts do not vary greatly between Fridays and Monday-Thursdays, and between Saturdays and Sundays. We believe that this assumption is valid, with two important exceptions. The first is visits to bars, discos, and shebeens. The latest Demographic and Health Survey conducted in South Africa shows that high alcohol consumption is far more common at weekends than on weekdays in Western Cape [24]. In this context, weekend is likely to refer to Friday and Saturday nights, rather than Saturdays and Sundays. We therefore believe that we have (potentially greatly) underestimated the amount of contact hours in bars in Western Cape. For this reason, we do not compare data on bars between the two sites. The second exception is visits to churches, which are likely to be more common on Sundays than on other days. Using an alternative weighting scheme, we show that we may have slightly overestimated the number of adult/youth and child contact hours in churches for both sites, but that differences between countries are robust to the choice of weights (Additional file 1: Table S4).

Finally, data were collected using a retrospective questionnaire, and not directly recorded or collected using prospective diaries. Recall bias is unlikely to be a large problem for this study, as data were collected on locations visited during the day before the interview only. Over short time periods, there is little difference in reported numbers of contacts using retrospective vs prospective study designs [25]. It is possible that some misreporting occurred through social desirability bias however, and respondents may have under-reported visits to or time spent in bars or clinics, and over-reported visits to churches or workplaces.

Data on indoor contacts by location type have previously been collected in a township near Cape Town, South Africa [15, 16]. In line with our results for Western Cape, the highest proportion of time was spent, and highest number of contacts were met, in workplaces (50 % of time, and 32 % of contacts, excluding time and contacts in participants’ own households and in transport, and excluding participants aged under 20 years). Considering differences in sampling data collection methods, roughly comparable proportions of time and contacts also occurred in other households, schools, and shops. In contrast to our study, Wood et al found that only 0.07 % of time and 0.8 % of contacts occurred in clinics (vs 2.1 % of hours and 5.1 % of contacts in this study). Differences could be due to differences in sampling and data collection methods, or reflect local variation between the township [15, 16] and Western Cape as a whole.

## Conclusions

Given that only a low proportion of *M.tb.* transmission in high incidence settings is believed to occur within households [6, 7], there is a real need for a better understanding of where transmission occurs in different settings. We demonstrate that patterns of indoor contact can differ substantially between different sites in high *M.tb* incidence areas. In our study, the highest proportion of indoor adult contact hours with both adults/youths and children (outside participants’ homes) occurred in workplaces in Western Cape, and in churches in urban Zambia. Targeting public buildings with interventions to reduce transmission (e.g. increasing ventilation or screening for TB) may be one way to reduce *M.tb.* incidence, but local data should be used when designing interventions.

### Availability of data and materials

The dataset used in this study is publicly available from http://dx.doi.org/10.17037/DATA.28 The questionnaire and further details of the sampling and interview methods are available in Dodd 2015 [20].

## Additional files

Additional file 1: **Additional methods and results.** (PDF 469 kb)

Additional file 2: **Completed Strobe checklist for cross-sectional studies.** (PDF 315 kb)
