# Supplementary material for: Comparison of indoor contact time data in Zambia and Western Cape, South Africa suggests targeting of interventions to reduce Mycobacterium tuberculosis transmission should be informed by local data
Source: BMC Infect Dis. 2016 Feb 9;16:71. doi: 10.1186/s12879-016-1406-5 (PMC4746903; doi:10.1186/s12879-016-1406-5)
Supplement: Supplementary file 1 — Additional methods and results. (PDF 469 kb) [file 12879_2016_1406_MOESM1_ESM.pdf]

	Western Cape	Zambia	Total
**Sunday**	195 (15.4%)	368 (19.0%)	563 (17.5%)
**Monday**	271 (21.3%)	347 (17.9%)	618 (19.2%)
**Tuesday**	281 (22.1%)	349 (18.0%)	630 (19.6%)
**Wednesday**	278 (21.9%)	307 (15.8%)	585 (18.2%)
**Thursday**	241 (19.0%)	375 (19.3%)	616 (19.2%)
**Friday**	4 (0.3%)	99 (5.1%)	103 (3.2%)
**Saturday**	0 (0.0%)	96 (4.9%)	96 (3.0%)

Table S1: Day of week that data were collected for, by site

		Western Cape	Zambia	p-value
**Mean number of adults/youths present per visit to: (Low estimate)**	**Other homes**	1.55 (0.96 - 2.14)	2.65 (2.00 - 3.30)	**0.02**
	**Shops**	10.43 (8.09 - 12.78)	5.07 (3.89 - 6.25)	**0.0001**
	**Churches**	18.51 (16.95 - 20.07)	16.58 (15.50 - 17.67)	**0.05**
	**Bars**	*13.50 (11.07 - 15.92)*	15.17 (13.77 - 16.56)	
	**Schools**	16.80 (14.41 - 19.20)	17.17 (15.09 - 19.24)	0.8
	**Clinics**	18.33 (15.70 - 20.96)	15.89 (14.16 - 17.63)	0.1
	**Hairdressers**	1.93 (-0.23 - 4.09)	4.91 (2.53 - 7.28)	0.07
	**Work**	9.36 (8.12 - 10.61)	10.16 (8.21 - 12.10)	0.5
	**Other**	11.78 (8.42 - 15.14)	8.65 (7.17 - 10.14)	0.09
**Mean number of adults/youths present per visit to: (High estimate)**	**Other homes**	6.82 (5.72 - 7.93)	9.00 (7.63 - 10.37)	**0.02**
	**Shops**	25.22 (19.54 - 30.89)	13.08 (10.57 - 15.60)	**0.0002**
	**Churches**	43.20 (38.94 - 47.46)	38.83 (36.19 - 41.47)	0.09
	**Bars**	*30.49 (24.32 - 36.66)*	34.47 (31.02 - 37.91)	
	**Schools**	39.36 (33.45 - 45.27)	40.12 (34.93 - 45.32)	0.8
	**Clinics**	42.99 (36.15 - 49.84)	36.86 (32.38 - 41.34)	0.1
	**Hairdressers**	5.93 (3.77 - 8.09)	12.21 (7.01 - 17.42)	**0.03**
	**Work**	21.99 (19.27 - 24.70)	23.15 (18.78 - 27.53)	0.7
	**Other**	27.78 (19.85 - 35.72)	20.61 (17.10 - 24.13)	0.1
**Mean number of children present per visit to: (Low estimate)**	**Other homes**	0.55 (0.19 - 0.91)	0.75 (0.35 - 1.16)	0.5
	**Shops**	5.11 (3.09 - 7.13)	2.14 (1.33 - 2.94)	**0.01**
	**Churches**	12.56 (8.36 - 16.76)	11.88 (10.18 - 13.58)	0.8

	**Bars**	*0.92 (-0.25 - 2.09)*	1.28 (0.72 - 1.85)	
	**Schools**	1.43 (0.13 - 2.74)	4.69 (2.39 - 6.98)	**0.02**
	**Clinics**	12.64 (8.67 - 16.62)	10.52 (7.83 - 13.21)	0.4
	**Hairdressers**	0.00*	1.47 (0.23 - 2.72)	-
	**Work**	1.80 (0.97 - 2.63)	2.38 (1.19 - 3.56)	0.4
	**Other**	2.11 (0.17 - 4.05)	4.12 (2.49 - 5.76)	0.1
**Mean number of children present per visit to: (Low estimate)**	**Other homes**	5.38 (4.68 - 6.08)	5.82 (4.97 - 6.68)	0.4
	**Shops**	13.74 (9.41 - 18.06)	7.95 (6.35 - 9.56)	**0.01**
	**Churches**	28.46 (17.87 - 39.04)	28.59 (24.72 - 32.47)	1.0
	**Bars**	*5.58 (3.35 - 7.80)*	6.24 (5.15 - 7.34)	
	**Schools**	6.95 (4.17 - 9.72)	14.24 (9.25 - 19.24)	**0.01**
	**Clinics**	29.77 (20.96 - 38.59)	25.12 (19.01 - 31.23)	0.4
	**Hairdressers**	4.00*	5.86 (3.98 - 7.74)	0.05
	**Work**	7.73 (5.97 - 9.49)	8.34 (5.86 - 10.83)	0.7
	**Other**	8.35 (4.10 - 12.60)	12.14 (8.53 - 15.75)	0.2

Table S2: Mean number of adults and youths (>12 years) and children (5-12 years) present in building by building type and site. Low and High estimates assume that the number of adults/youths and children in buildings were equal to category lower bounds and upper bounds (see methods for details). p-values in bold indicate significance at the 95% level.*Confidence intervals could not be calculated due to low numbers. Estimates for bars in Western Cape are considered to be unreliable (see discussion), and therefore p-values are not shown.

		Western Cape	Zambia	p-value
**Mean number of adult/youth contact hours per adult per day in: (Low estimate)**	**Other homes**	1.38 (0.60 - 2.15)	1.67 (0.85 - 2.49)	0.6
	**Shops**	1.78 (0.71 - 2.85)	1.67 (0.86 - 2.47)	0.9
	**Churches**	2.55 (1.33 - 3.78)	5.94 (4.23 - 7.65)	**0.002**
	**Bars**	*1.35 (0.47 - 2.22)*	3.69 (2.59 - 4.78)	
	**Schools**	3.29 (2.13 - 4.45)	3.58 (2.46 - 4.69)	0.7
	**Clinics**	1.04 (0.50 - 1.58)	1.15 (0.65 - 1.65)	0.8
	**Hairdressers**	0.09 (0.00 - 0.21)	0.28 (0.02 - 0.53)	0.2
	**Work**	10.08 (7.23 - 12.93)	5.42 (3.57 - 7.27)	**0.01**
	**Other**	1.43 (0.40 - 2.46)	1.96 (1.08 - 2.83)	0.4
	**Total**	22.96 (18.85 - 27.07)	25.29 (21.64 - 28.95)	0.4
**Mean number of adult/youth contact hours per adult per day in: (High estimate)**	**Other homes**	6.05 (3.98 - 8.12)	5.15 (3.03 - 7.27)	0.5
	**Shops**	4.22 (1.69 - 6.75)	3.97 (2.11 - 5.83)	0.9
	**Churches**	5.95 (3.07 - 8.84)	13.90 (9.87 - 17.94)	**0.002**
	**Bars**	*3.09 (1.03 - 5.15)*	8.44 (5.90 - 10.97)	
	**Schools**	7.76 (5.03 - 10.49)	8.39 (5.75 - 11.04)	0.7
	**Clinics**	2.43 (1.16 - 3.71)	2.68 (1.50 - 3.86)	0.8
	**Hairdressers**	0.19 (0.00 - 0.42)	0.61 (0.07 - 1.16)	0.2
	**Work**	23.86 (17.35 - 30.36)	12.35 (8.15 - 16.55)	**0.004**
	**Other**	3.34 (0.91 - 5.76)	4.60 (2.49 - 6.71)	0.4
	**Total**	56.82 (47.21 - 66.44)	59.99 (51.43 - 68.55)	0.6
	**Other homes**	0.61 (0.14 - 1.09)	0.40 (0.20 - 0.61)	0.4
	**Shops**	0.91 (0.31 - 1.50)	0.64 (0.32 - 0.96)	0.4

**Mean number of child contact hours per adult per day in: (Low estimate)**	**Churches**	1.72 (0.68 - 2.76)	4.19 (2.84 - 5.54)	**0.005**
	**Bars**	*0.04 (0.00 - 0.10)*	0.21 (0.09 - 0.32)	
	**Schools**	0.03 (0.00 - 0.08)	0.86 (0.24 - 1.48)	**0.01**
	**Clinics**	0.70 (0.24 - 1.17)	0.76 (0.33 - 1.18)	0.9
	**Hairdressers**	0.00 (0.00 - 0.00)	0.13 (0.00 - 0.29)	0.1
	**Work**	2.02 (0.98 - 3.06)	1.01 (0.39 - 1.62)	0.1
	**Other**	0.18 (0.02 - 0.33)	0.94 (0.28 - 1.59)	**0.03**
	**Total**	6.20 (4.60 - 7.80)	9.06 (6.99 - 11.13)	**0.03**
**Mean number of child contact hours per adult per day in: (High estimate)**	**Other homes**	5.06 (3.40 - 6.71)	2.79 (1.75 - 3.83)	**0.02**
	**Shops**	2.20 (0.79 - 3.61)	1.84 (1.08 - 2.59)	0.7
	**Churches**	3.91 (1.44 - 6.39)	10.06 (6.81 - 13.31)	**0.004**
	**Bars**	*0.40 (0.18 - 0.62)*	1.16 (0.78 - 1.54)	
	**Schools**	0.74 (0.50 - 0.99)	2.59 (1.12 - 4.06)	**0.02**
	**Clinics**	1.65 (0.56 - 2.73)	1.82 (0.81 - 2.82)	0.8
	**Hairdressers**	0.11 (0.00 - 0.21)	0.33 (0.00 - 0.66)	0.2
	**Work**	8.62 (6.04 - 11.21)	3.50 (1.98 - 5.02)	**0.001**
	**Other**	0.92 (0.45 - 1.39)	2.56 (0.90 - 4.21)	0.1
	**Total**	23.55 (19.50 - 27.59)	26.41 (21.42 - 31.40)	0.4

Table S3: Mean number of adult and youth (>12 years) and child (5-12 years) contact hours per adult per day by building type and site. Low and High estimates assume that the number of adult/youths and children in buildings were equal to category lower bounds and upper bounds (see methods for details). p-values in bold indicate significance at the 95% level. Estimates for bars in Western Cape are considered to be unreliable (see discussion), and therefore p-values are not shown.

	Default weights			Alternative weights		
	**Western Cape**	**Zambia**	**p-value**	**Western Cape**	**Zambia**	**p-value**
**Proportion of respondents who visited a church the day before the interview**	5.2% (3.3% - 8.1%)	11.9% (9.3% - 15.2%)	0.001	3.6% (2.3% - 5.6%)	10.2% (8.0% - 13.1%)	**<0.0001**
**Mean visit duration (hours)**	2.68 (2.31 - 3.04)	2.75 (2.47 - 3.02)	0.8	2.74 (2.34 - 3.15)	2.74 (2.45 - 3.02)	0.98
**Mean number of adults/youths present per visit**	26.60 (24.47 - 28.73)	23.98 (22.50 - 25.46)	0.05	27.00 (25.17 - 28.83)	22.97 (21.43 - 24.51)	**0.001**
**Mean number of children present per visit**	18.25 (12.36 - 24.15)	17.60 (15.30 - 19.89)	0.8	18.28 (12.47 - 24.09)	15.80 (13.49 - 18.11)	0.43
**Mean adult/youth contact hours per adult per day**	3.67 (1.91 - 5.43)	8.57 (6.12 - 11.02)	0.002	2.65 (1.35 - 3.95)	7.03 (5.06 - 8.99)	**0.0004**
**Mean child contact hours per adult per day**	2.49 (1.00 - 3.98)	6.16 (4.21 - 8.12)	0.004	1.68 (0.67 - 2.69)	4.82 (3.28 - 6.35)	**0.001**

Table S4: Effect of using alternative weighting system on estimates for church visits, by site. The default weighting scheme weights observations according to whether the respondent was asked about building visits on a weekday or a weekend. The alternative weighting scheme weights observations according to whether the respondent was asked about building visits on a Sunday or on any other day of the week.

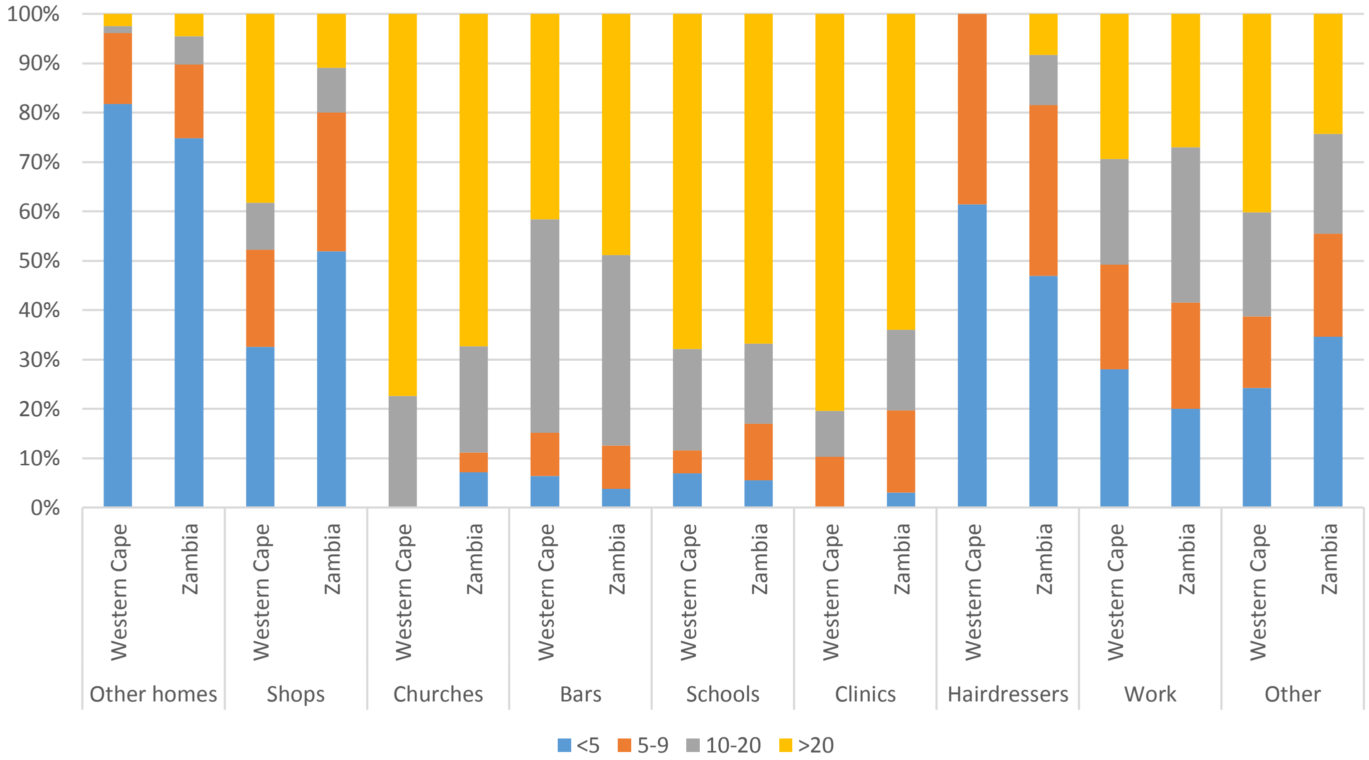

Figure S1. Distribution of reported numbers of adults/youths in building per visit, by building type and site

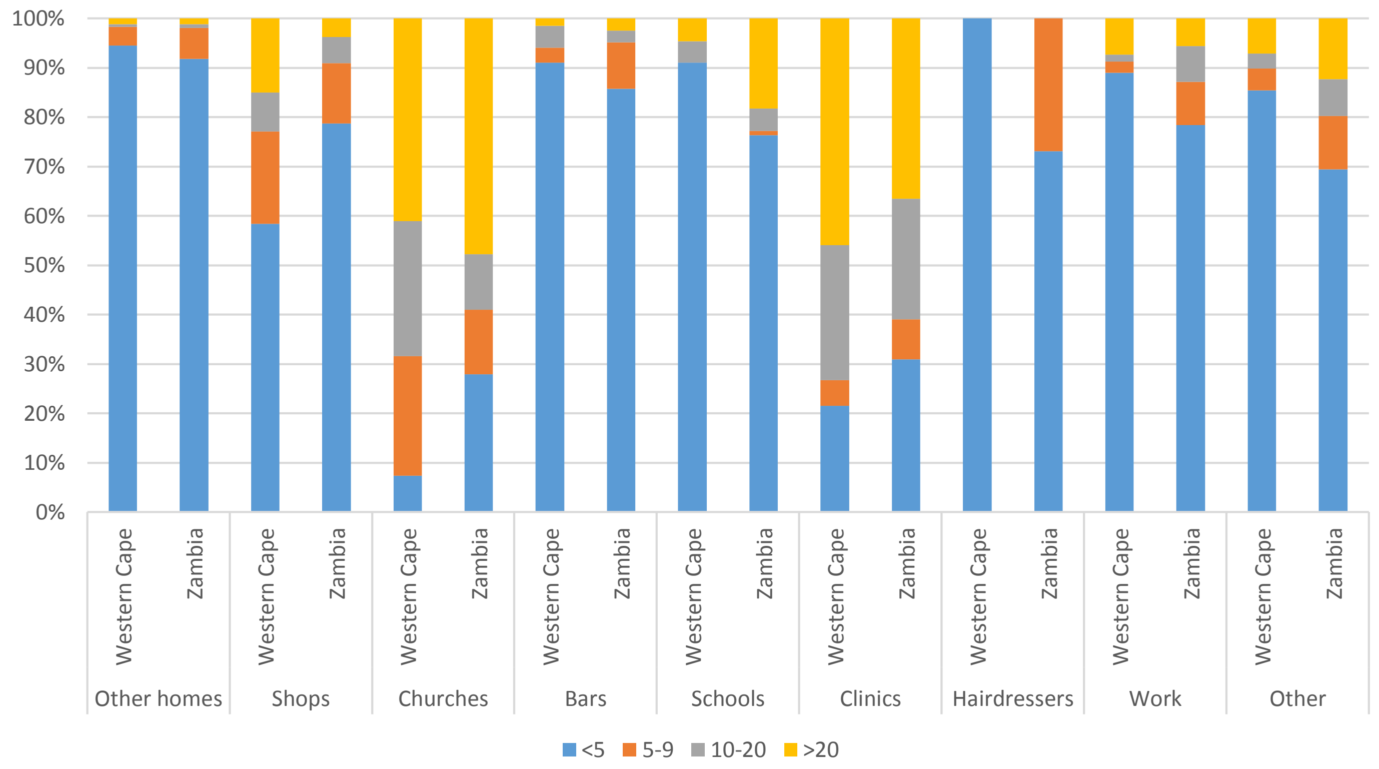

Figure S2. Distribution of reported numbers of children (aged 5-12 years) in building per visit, by building type and site

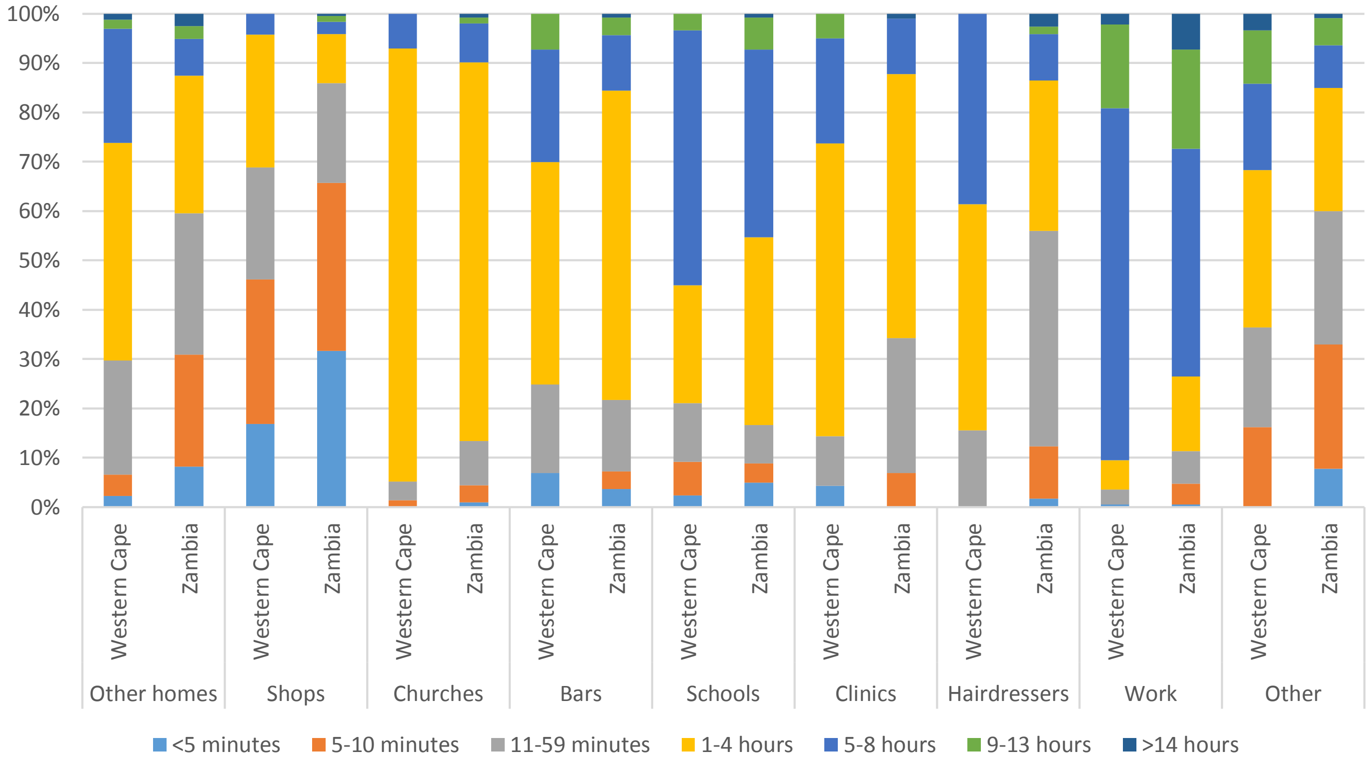

Figure S3. Distribution of reported time spent in building per visit, by building type and site
